# Bowel Wall Thickness Cutoff Value for Assessing Inflammatory Bowel Disease Activity Using Intestinal Ultrasonography in Children

**DOI:** 10.1093/ibd/izaf298

**Published:** 2025-12-08

**Authors:** Laura Räisänen, Ray Lang, Michael Couper, Peter Lewindon

**Affiliations:** Tampere Center for Child, Adolescent, and Maternal Health Research, Faculty of Medicine and Health Technology, Tampere University , Tampere, Finland; Department of Paediatrics, Wellbeing Services County of Pirkanmaa, Tampere University Hospital, Tampere, Finland; Department of Gastroenterology, Hepatology, and Liver Transplant, Queensland Children’s Hospital, Brisbane, Queensland, Australia; Department of Gastroenterology, Hepatology, and Liver Transplant, Queensland Children’s Hospital, Brisbane, Queensland, Australia; Paediatric Medicine, Gastroenterology, Women and Children’s Hospital, Adelaide, South Australia, Australia; Department of Gastroenterology, Hepatology, and Liver Transplant, Queensland Children’s Hospital, Brisbane, Queensland, Australia; Queensland Children’s Medical Research Institute, University of Queensland, Brisbane, Queensland, Australia

**Keywords:** Crohn’s disease, ulcerative colitis, noninvasive tool, diagnostic methods, pediatric

## Abstract

**Background and Aims:**

Intestinal ultrasonography (IUS) is a noninvasive tool for assessing bowel inflammation. In adults, a bowel wall thickness (BWT) cutoff of 0.30 cm is used to indicate inflammation, but children do not have a widely accepted value. We propose an optimal BWT cutoff value for children.

**Methods:**

We performed 144 IUS examinations during 2019-2024 in 133 children within 30 days before to 7 days after colonoscopy. Assessed values for the BWT from the terminal ileum to the rectum were paired with colonoscopy findings at each segment (*n* = 809 pairs). The cutoff value for detecting inflammation was explored with receiver operating characteristic (ROC) analysis.

**Results:**

In children ≥6 years old IUS demonstrated excellent accuracy for detecting moderate/severe inflammation (area under the curve [AUC] 0.907) but poor accuracy for mild inflammation (AUC 0.690) or in children <6 years old (AUC, 0.667). The adult cutoff (0.30 cm) missed 32% of inflammation in children. In children ≥6 years old, a BWT cutoff of 0.27 cm detected 85% of moderate/severe and 43% of mild inflammation. Lower cutoff values (0.24 cm) were more optimal for girls and children weighing <40 kg.

**Conclusions:**

Although IUS is an excellent tool for detecting moderate/severe bowel inflammation, this method showed limited accuracy in children <6 years old. The adult BWT cutoff missed over 30% of bowel inflammation in children. BWT cutoff of 0.27-0.30 cm in boys and 0.23-0.25 cm in girls and/or children <40 kg indicated active gut inflammation.

Key Messages *What is already known?*

A bowel wall thickness (BWT) cutoff of 0.30 cm is used to indicate inflammation in adults, but no established value is available for children.

*What is new here?*

The adult BWT cutoff of 0.30 cm missed over 30% of bowel inflammation in children. A lower cutoff value of 0.27-0.30 cm was more effective in children ≥6 years old and even lower values (0.23-0.25 cm) are suggested for girls and children weighing <40 kg. The use of IUS showed limited accuracy in children <6 years old.

*How can this study help patient care?*

Using pediatric cutoff values with cutoffs selecte according to sex and weight specific physiological differences can improve diagnostic accuracy and ensure more appropriate and timely treatments in children ≥6 years old.

## Introduction

Pediatric inflammatory bowel diseases (PIBD) is increasing worldwide.^1^ Treatment goals now focus on altering the disease course, reducing relapses and surgeries, improving quality of life, and ultimately finding curative treatments and effective prevention strategies. To accomplish these aims, reliable and accessible tools that provide prompt information on disease state and responses to treatment are essential. Fecal calprotectin (FC) measurement has been a well-accepted tool for assessing disease activity but does not provide data on disease location and severity. Therefore, intestinal ultrasonography (IUS) is now well accepted as a significant complementary tool. This procedure is noninvasive, accessible, repeatable, and capable of detecting inflammation and early treatment response within 4-8 weeks.^2^ In adults, BWT >0.30 cm is accepted as a reliable measure of bowel wall inflammation that has been validated by colonoscopy.^3^^,^^4^

In children, a bowel wall thickness (BWT) cutoff of 0.25-0.30 cm is frequently used based on literature describing adult patients, but validated values for children are lacking.^5^ In a study from 1997 involving 38 children 4-18 years old (9 with ulcerative colitis [UC]; 21 with Crohn's disease [CD]), a BWT cutoff of 0.25 cm was proposed for the terminal ileum and 0.20 cm for the colon.^6^ However, the sex and size of the children and disease severity observed in colonoscopy were not addressed in this study. Increased BWT to >0.30 cm has been reported to have high sensitivity for severe disease, but identifying mild inflammation remains difficult due to variable sensitivity and specificity.^7^ Moreover, cutoff values for adults or older children may miss inflammation in younger children. It may also be necessary to stratify threshold values by disease subtype (UC vs CD), sex, and age, as current adult-based reference ranges may not sufficiently account for the physiological differences present in pediatric populations.

In this study we retrospectively utilized the IUS database of a single institution to propose more optimal BWT cutoff values that will detect bowel inflammation confirmed by macroscopic and histologic findings in children. We also determined whether cutoff values would vary by bowel segment, IBD subtype (CD vs UC), and age group (including children younger than 6 years with VEO-IBD).

## Method

### Intestinal ultrasound (IUS)

This is a retrospective study using hospital records. At our IBD clinic in the Queensland Children’s Hospital, IUS has been actively used to compliment endoscopy, laboratory tests (including FC), and Paeditaric Crohn’s Disease Activity Index (PCDAI)/Paediatric Ulcerative Colitis Activity Index (PUCAI) in diagnosing and monitoring inflammation. For instance, 432 children underwent 824 IUS scans between September 2019 and 2024. All IUS examinations were performed by a sole operator (PL, certified by international bowel ultrasound group (IBUS) and Gastroenterology network of intestinal ultrasound (GENIUS)) using Aixplorer SuperSonic Imagine (RAP2102LM-SSI) with a linear probe (SL10-2, 2-10 MHz, 38 mm).

IUS images were extracted from the hard drive of the ultrasound machine. The images included patient details (name and date of birth), examination date, bowel segment annotations (terminal ileum, caecum, ascending, transverse, descending, sigmoid, and rectum) and BWT (cm) as a two-digit continuous variable. Due to volume of data, images were processed using Python 3.7. Optical character recognition software (Tesseract 4 for windows) was used to extract site and BWT data from the saved IUS images into structured tables. To ensure data accuracy and to get a (conclusive) BWT value used for making clinical decision, the extracted BWT values from each segment were cross-referenced with BWT values reported by the operator in the clinical notes, assessable through the Integrated Electronic Medical Record (iEMR). No remarkable discrepancies between BWT marked in the obtained images and BWT reported in the iEMR were observed.

### Colonoscopy

PIBD diagnosis and disease phenotypes were established by pediatric gastroenterologists based on standardised criteria.^8^ Data on demography, date of diagnosis, and colonoscopy findings (including Mayo or simple endoscopic scores (SES-CD) scores, examination dates, and colonoscopy images) were obtained from the iEMR. Colonoscopy with histology were used as the reference standard for confirming colonic inflammation.

Overall colonic severity is usually categorised using endoscopic Mayo scores (remission (0), mild (1), moderate (2), and severe (3)) for UC and total SES-CD (remission (0-2), mild (3-6), moderate (7-16), and severe (≥16) as a sum variable of segmental SES-CD.^9^ The segmental SES-CD is calculated based on the presence and size of ulcers (0-3), extent of affected surface at each segment (0-3), and presence of narrowing (0-3). While IUS can be used to detect colon inflammation prior to diagnostic colonoscopy, it could not be used to determine IBD phenotype (UC or CD). Therefore, overall inflammation at each segment of the colon in this study was graded using macroscopic scores marked by endoscopist during colonoscopy (segmental Mayo for children with UC/segmental SES-CD for children with CD), along with histologic findings.

In children with UC and segmental Mayo 0 at colonoscopy, 28/142 (20%) segments showed inflammation at histology. Moreover, of segments with Mayo 1, 8/97 (8%) showed normal histological findings. Regardless of IBD phenotype, the combination of histology and macroscopic scoring were used to classify colonic inflammation at each segment as: **absent** (Mayo 0-1/SES-CD 0-2 without ulcers, confirmed by normal histology); **mild** (Mayo 0-1 with active inflammation on histology/SES-CD 0-2 with/without ulcers and with confirmed inflammation at histology, or SES-CD ≥3 with the presence of inflammatory lesions (erythema, oedema, and aphthae) but without ulceration); and **moderate/severe** (Mayo 2-3/SES-CD ≥7 with the presence of superficial or deep ulcers).^10^ For analysis, these classifications were also dichotomised as normal (no inflammation) vs inflamed (mild, moderate, or severe inflammation).

### Variables and outcomes

Colonoscopy dates were aligned with IUS dates. To minimize the impact of possible treatment-related mucosal changes between procedures, only BWT obtained within 30 days before or 7 days after colonoscopy were included. In 253/809 (31%) IUS scans with available colonoscopy pair, the BWT measurements were stated as “normal” in the medical record (i.e., the bowel wall was not prominent, the stratification looked normal, and the estimated BWT was <0.20-0.25 cm according to the operator),^11^ but no images with corresponding BWT measurements were available in the extracted images. In these 253 scans stated as “normal”, imputation was needed to perform receiver operating characteristic (ROC)-curve analysis. Of these imputation-requiring scans, 180/253 (71%) values were imputed using available measured BWT values from adjacent or other segments of colon from the same patient at the same IUS session, also reported as “normal” in the operator’s clinical notes. In the remaining 73/253 (29%) imputation-requiring scans, no adjacent values stated as “normal” were available. Therefore, in the latter case the imputation was performed using the mean BWT of children with matching age and sex from the first 180 imputation (Table S1).

If the colonoscopy data were available but no BWT images were extracted nor commented in the iEMR (i.e., not stated as “normal” by the operator), these colonoscopy findings were excluded from our study. This resulted in 809 paired BWT–bowel segment finding from 144 IUS–colonoscopy pairs in 133 children. Optimal BWT cutoffs for detecting inflammation were then determined based on highest sensitivity and specificity.

### Statistical analysis

Demographics were reported as frequencies (%) for categorical variables and as mean ± standard deviation (SD) or median (IQR) for continuous variables. Analyses were conducted per IUS–colonoscopy pair. Pearson’s chi-square for categorical and independent t-tests for continuous variables were used, with *P *< 0.05 considered significant. Missing data are noted in tables. When comparing means of continuous variables, independent t-test with two-sided p-values or one was ANOVA was used.

BWT cutoff values were determined using Receiver Operating Characteristic (ROC) analysis, with inflammation detected at colonoscopy and confirmed by histology as reference. To identify a generalisable cutoff value, all BWT–bowel segment pairs from different segments (including terminal ileum) were analysed collectively. Given the differences between UC (mucosal) and CD (transmural) inflammation, sub-analyses were performed by IBD subtype in children ≥6 years. Children <6 years were analysed separately. Additional analyses assessed BWT cutoff for boys vs girls, for children weighed <40 kg vs ≥40 kg, and for children <12 years vs ≥12 years. The accuracy of BWT for detecting inflammation was evaluated using area under the curve (AUC) values, classified as excellent (AUC ≥0.9), good (0.8 ≤ AUC <0.9), fair (0.7≤ AUC <0.8), poor (0.6≤ AUC <0.7) or fail (0.6 ≤ AUC <0.5).^12^ Statistical analyses were performed using SPSS Statistics 29.0.0.0.

## Results

This study involved 130 children with PIBD (72 UC including 5 PSC-UC and 4 IBDU; 48 CD; 10 VEO-IBD) and 3 children that did not obtain nor develop PIBD diagnosis in subsequent follow-up. Among the children with PIBD, 14 with UC and 3 with CD had two IUS-colonoscopy examination pairs, resulting in 150 IUS-colonoscopy pairs (147 from children with PIBD and 3 from children without PIBD). Characteristics of the 147 IUS-colonoscopy pairs from 130 children with PIBD are presented in Table 1.

**Table 1.** Background characteristics of 147 intestinal ultrasonography (IUS)–colonoscopy pairs (without imputation) from children with pediatric inflammatory bowel diseases.

Baseline clinical characteristics of IUS-colonoscopy pairs	Type of colitis			**Total** , **No. 147**
	UC, No. 86	CD, No. 51	VEO-IBD, No. 10	
**Age of IUS, mean (±SD) years**	13.2 (±2.3)	12.7 (±2.4)	4.2 (±1.3)	12.4 (±3.2)
**Sex, n (%)**				
** Boys**	43 (50)	28 (55)	4 (40)	75 (51)
** Girls**	43 (50)	23 (45)	6 (60)	72 (49)
**Purpose of IUS-colonoscopy examination, n (%)**				
** Diagnostic**	58 (67)	35 (69)	9 (90)	102 (69)
** Follow-up**	28 (33)	16 (31)	1 (10)	45 (31)
**Time difference between IUS and colonoscopy, median (IQR) days**				
** Date IUS ≤ date colonoscopy, n = 62 + 39 + 7**	2 (1-7)	7 (3-15)	1 (1-5)	4 (1-11)
** Date IUS > date colonoscopy, n = 24 + 12 + 3**	2 (1-3)	1 (1-2)	1 (1-5)	2 (1-3)
**No. of available measured IUS-colonoscopy pairs at different segments, n (%)**				
** Terminal ileum (36 inflamed, 19 normal)**	17 (20)	36 (71)	2 (20)	55 (37)
** Caecum (63 inflamed, 9 normal)**	37 (43)	30 (59)	5 (50)	72 (49)
** Ascending (61 inflamed, 18 normal)**	39 (45)	33 (65)	7 (70)	79 (54)
** Transverse (64 inflamed, 21 normal)**	46 (53)	33 (65)	6 (60)	85 (58)
** Descending (83 inflamed, 16 normal)**	63 (73)	29 (57)	7 (70)	99 (67)
** Sigmoid (96 inflamed, 18 normal)**	71 (83)	36 (71)	7 (70)	114 (78)
** Rectum (43 inflamed, 2 normal)**	33 (38)	10 (20)	2 (20)	45 (31)
**TOTAL (446 inflamed, 103 normal), n (%)**	306/602 (51)	207/357 (58)	36/70 (51)	549/1029 (53)
**Measured BWT of the 446 inflamed segments (including colon and terminal ileum) before/after colonoscopy, mean (±SD) cm**				
** Before colonoscopy (n = 171 + 117 + 25)**	0.34 (±0.11)	0.36 (±0.13)	0.29 (±0.14)	0.34 (0.12)
** After colonoscopy (n = 91 + 32 + 10)**	0.35 (±0.10)	0.35 (±0.16)	0.33 (±0.14)	0.35 (0.12)
***P-value***	*0.375*	*0.668*	*0.398*	*0.611*
**Measured BWT at each segment when inflamed, mean (±SD) cm**				
** Terminal ileum, n = 36 (4 + 30 + 2)**	0.29 (±0.04)	0.39 (±0.15)	0.25 (±0.04)	0.37 (±0.15)
** Caecum, n = 63 (32 + 26 + 5)**	0.32 (±0.09)	0.39 (±0.15)	0.32 (±0.16)	0.35 (±0.13)
** Ascending, n = 61 (33 + 22 + 6)**	0.30 (±0.11)	0.32 (±0.12)	0.29 (±0.17)	0.31 (±0.12)
** Transverse, n = 64 (39 + 19 + 6)**	0.33 (±0.11)	0.34 (±0.14)	0.29 (±0.15)	0.33 (±0.12)
** Descending, n = 83 (57 + 19 + 7)**	0.34 (±0.10)	0.34 (±0.10)	0.33 (±0.12)	0.34 (±0.10)
** Sigmoid, n = 96 (64 + 25 + 7)**	0.34 (±0.11)	0.32 (±0.09)	0.27 (±0.15)	0.33 (±0.11)
** Rectum, n = 43 (33 + 8 + 2)**	0.41 (±0.12)	0.42 (±0.17)	0.36 (±0.20)	0.41 (±0.13)
**Measured BWT when normal, mean (±SD)**	(n = 44)	(n = 58)	(n = 1)	(n = 103)
	0.23 (±0.09)	0.25 (±0.09)	0.08	0.24 (±0.09)
**Bowel segments stated as “normal” in the medical record without available measured BWT, n (%)**	(n = 129)	(n = 96)	(n = 18)	(n = 243)
** Normal colonoscopy (n = 78 + 58 + 2)**	78 (60)	58 (60)	2 (11)	138 (57)
** Inflamed at colonoscopy (n = 51 + 38 + 16)**	51 (40)	38 (40)	16 (89)	105 (43)
**Degree of inflammation at different segments based on colonoscopy and histology, n (%)**				
** Absent**	122 (28)	116 (38)	3 (6)	241 (30)
** Mild**	117 (27)	102 (34)	24 (44)	243 (31)
** Moderate/Severe**	196 (45)	85 (28)	27 (50)	308 (39)
**TOTAL available segments at colonoscopy, n (%)**	435/602 (72)	303/357 (85)	54/70 (77)	792/1029 (77)

IUS= Intestinal Ultrasound; BWT=Bowel wall thickness; IQR=Interquartile range.

Three children with normal diagnostic colonoscopy had 7 BWT measurements (1 from terminal ileum, caecum, and transverse colon; 2 from descending and sigmoid colon) with mean BWT of 0.23 (±0.09) cm, and 10 scans stated as “normal” without available measured BWT.

Accurate BWT measurement of the rectum is technically challenging due to its position behind the bladder, limiting available measured IUS–colonoscopy pairs to 47/150 (31%). Moreover, the terminal ileum was not reached in all colonoscopies, reducing measured ileal IUS-colonoscopy pairs to 56/150 (37%). The sigmoid colon had the highest availability, with 116/150 pairs (77%). In total, 556 bowel segments with measured BWT (549 from children with PIBD and 7 from children without IBD) and 253 bowel segments stated as “normal” in IUS examination (243 from children with PIBD and 10 from children without IBD) had corresponding colonoscopy pairs, generating 809 pairs of BWT-colonoscopy bowel segment examinations.

Of all 809 scans, 281 (34.7%) were done the day before (n = 98), on the same day (n = 95), or the day after (n = 88) colonoscopy. IUS was performed within median 4 (IQR 1–12) days before colonoscopy in 111 cases (596 bowel segments) or within median 2 (IQR 1–3) days after colonoscopy in 39 cases (213 bowel segments). Only 51 scans (0.06%) were performed within 3-6 days after colonoscopy, reducing the effect of any possible treatment initiated usually a day or two after colonoscopy. Furthermore, in inflamed bowel segments, no BWT differences were seen in IUS performed before or after colonoscopy (0.34 ± 0.12 cm vs 0.35 ± 0.12 cm, *P *= 0.611, Table 1). Therefore, separate analyses based on timing of IUS were not conducted.

### Bowel wall thickness and degree of inflammation

**Figure 1.**
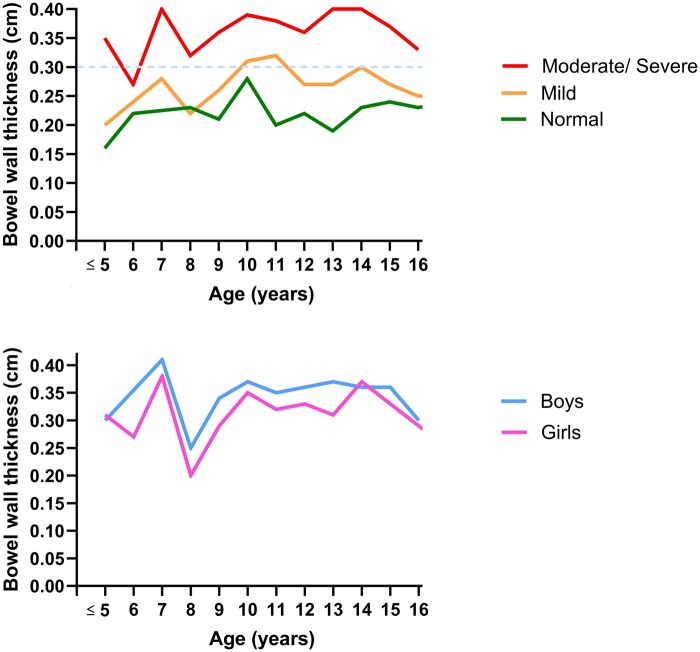
Mean bowel wall thickness (BWT) at different degree of inflammation (upper) and in boys or girls with any degree of inflammation (lower).

Mean BWT for different degree of inflammation across all 556 measurements (549 from children with PIBD and 7 from children without IBD diagnosis) of paired colonoscopy-IUS segments are provided in Table 2. BWT showed a correlation with the degree of inflammation, but not with age (Figure 1). In the presence of bowel inflammation (either mild or moderate/severe), boys had thicker mean BWT than girls (0.35 ± 0.13 cm vs 0.33 ± 0.11 cm, *P *= 0.046). The bowel wall of children with CD was thicker than in those with UC during mild inflammation (Table 2), but no significant differences were seen when measuring BWT during inflammation (combining both mild and moderate/severe) in general (0.36 ± 0.13 in CD vs 0.34 ± 0.11 in UC, *P *= 0.200).

**Table 2.** Measured mean bowel wall thickness (BWT) in inactive, mild, moderate, and severe bowel inflammation.

Mean bowel wall thickness (BWT) ±SD, cm	Degree of inflammation		
	Absent (n = 110)	Mild (n = 162)	Moderate/severe (n = 163)
**Sex**			
**Boys, n = 68 + 94 + 146**	0.24 (±0.08)	0.30 (±0.10)	0.39 (±0.13)
**Girls, n = 42 + 68 + 138**	0.23 (±0.11)	0.26 (±0.09)	0.36 (±0.10)
***P-value***	*0.362*	***0.033***	*0.057*
**Diagnosis**			
**UC, n = 44 + 78 + 184**	0.23 (±0.09)	0.27 (±0.09)	0.37 (±0.10)
**CD, n = 58 + 71 + 78**	0.25 (±0.09)	0.31 (±0.10)	0.40 (±0.15)
***P-value***	*0.458*	***0.003***	*0.132*
**VEO-IBD, n = 1 + 13 + 22**	0.08	0.21 (±0.07)	0.35 (±0.14)
**No IBD, n = 7**	0.23 (±0.09)	n/a	n/a
**Purpose of IUS-colonoscopy examination**			
**Diagnostic, n = 80 + 119 + 205**	0.23 (±0.10)	0.29 (±0.10)	0.38 (±0.12)
**Follow-up, n = 30 + 43 + 79**	0.25 (±0.09)	0.27 (±0.09)	0.38 (±0.11)
***P-value***	*0.441*	*0.189*	*0.845*
**Site of IUS**			
**Terminal ileum, n = 20 + 19 + 17**	0.30 (±0.12)	0.30 (±0.11)	0.45 (±0.14)
**Caecum, n = 10 + 30 + 33**	0.31 (±0.07)	0.29 (±0.08)	0.40 (±0.14)
**Ascending, n = 18 + 27 + 34**	0.22 (±0.10)	0.26 (±0.12)	0.35 (±0.10)
**Transverse, n = 22 + 26 + 38**	0.21 (±0.07)	0.28 (±0.08)	0.37 (±0.13)
**Descending, n = 18 + 24 + 59**	0.22 (±0.06)	0.29 (±0.08)	0.36 (±0.10)
**Sigmoid, n = 20 + 27 + 69**	0.20 (±0.08)	0.26 (±0.08)	0.36 (±0.10)
**Rectum, n = 2 + 9 + 34**	0.21 (±0.11)	0.36 (±0.12)	0.42 (±0.13)
***P-value***	*0.162*	*0.668*	*0.424*
***TOTAL***	*0.24 (±0.09)*	*0.28 (±0.10)*	*0.38 (±0.12)*

Abbreviations: UC, Ulcerative colitis; CD, Crohn’s disease; VEO-IBD, Very-early onset inflammatory bowel diseases; SD, standard deviation.

*P*-values were obtained using independent sample t-test.

No significant BWT differences between right (caecum to ascending) and left (descending, sigmoid, and rectum) colon were seen during mild (0.27 ± 0.10 cm vs 0.29 ± 0.10 cm, *P *= 0.463) or moderate/severe inflammation (0.37 ± 0.12 cm vs 0.37 ± 0.11 cm, *P *= 0.956). In moderately/severely inflamed cases, the terminal ileum had thicker BWT than the colon (0.45 ± 0.14 cm vs 0.37 ± 0.11, *P *= 0.007). For cutoff value analysis, all BWT data from colonic segments (n = 709, including 500 measured and 209 imputed values) were combined, while terminal ileum measurements (n = 100, including 56 measured and 44 imputed values) were analysed separately.

### Cutoff values for different IBD phenotypes

#### Ulcerative colitis

Based on measured BWT, the commonly used cutoff of 0.30 cm detected 186/262 (71%) and missed 76/262 (29%) of colonoscopically confirmed inflamed segments (including both colon and ileum). In investigating BWT cutoff value for UC, paired IUS-colonoscopy (including imputed values) was available for 72 children with UC (389 paired segments from colon and 46 from terminal ileum) and 3 children without IBD (14 paired segments from colon and 3 from terminal ileum) – generating 403 paired IUS-colonoscopy segments from colon and 49 from terminal ileum. With good accuracy (AUC 0.826, 95% CI 0.782-0.869), a BWT cutoff value of 0.27 cm presented 67% sensitivity and 90% specificity for detecting any inflammation (Table 3). A suggested lower cutoff of 0.24 cm for girls (AUC 0.851, 95%CI 0.795-0.907) showed 78% sensitivity and 93% specificity. Since inflammation of terminal ileum (i.e., backwash ileitis) at colonoscopy was present in only 8/49 segments, further analysis of BWT cutoff for backwash ileitis in UC was not pursued.

**Table 3.** Optimal bowel wall thickness (BWT) cutoff values for detecting inflammation of Colon in different IBD phenotypes and degrees of inflammation.

		AUC	95% CI	Maximum Youden’s index	Proposed BWT cutoff value (cm)	Sensitivity (%)	Specificity (%)
			*Any degree of colon inflammation*				
**Boys**	**UC**	0.772	0.699-0.845	0.481	0.28	62	85
	**CD**	0.781	0.770-0.900	0.596	0.30	71	89
**Girls**	**UC**	0.851	0.795-0.907	0.709	0.24	78	93
	**CD**	0.835	0.695-0.867	0.628	0.24	71	92
**Both**	**UC**	0.826	0.782-0.869	0.564	0.27	67	90
	**CD**	0.804	0.751-0.856	0.558	0.27	68	88
**All ≥ 6 yrs**		0.815	0.782-0.848	0.552	0.27	67	88
**VEO-IBD**		0.667	0.526-0.808	0.420	0.22	55	87
			*Mild colon inflammation*				
**Boys**	**UC**	0.594	0.485-0.704	0.139	0.28	29	85
	**CD**	0.795	0.708-0.882	0.528	0.27	71	82
**Girls**	**UC**	0.674	0.560-0.789	0.395	0.23	50	90
	**CD**	0.670	0.541-0.799	0.473	0.24	55	92
**Both**	**UC**	0.666	0.593-0.739	0.283	0.23	52	77
	**CD**	0.738	0.664-0.811	0.466	0.27	59	88
**All ≥ 6 yrs**		0.690	0.638-0.742	0.311	0.27	43	88
**VEO-IBD**		0.512	0.309-0.714	0.367	0.22	50	87
			*Moderate or severe colon inflammation*				
**Boys**	**UC**	0.898	0.839-0.956	0.722	0.28	87	85
	**CD**	0.888	0.806-0.971	0.740	0.30	85	89
**Girls**	**UC**	0.925	0.877-0.972	0.846	0.24	92	93
	**CD**	0.905	0.825-0.984	0.802	0.24	88	92
**Both**	**UC**	0.916	0.880-0.952	0.765	0.27	87	90
	**CD**	0.884	0.826-0.941	0.682	0.30	77	92
**All ≥ 6 yrs**		0.907	0.879-0.935	0.728	0.27	85	88
**VEO-IBD**		0.781	0.639-0.924	0.459	0.22	59	87

Abbreviations: AUC, Area under the curve; CI, Confidence Interval; BWT, Bowel wall thickness; UC, Ulcerative colitis; CD, Crohn’s disease; VEO-IBD, Very-early onset inflammatory bowel diseases (with cutoff value for detecting inflammation at both colon and terminal ileum).

Using Mayo scoring only without histological results to confirm inflammation (i.e., Mayo 0 for absence of inflammation vs Mayo 1-3 for presence of inflammation) did not change the suggested general cutoff value of 0.27 cm (AUC 0.829, 95% CI 0.786-0.872; 68% sensitivity and 90% specificity) and a lower cutoff value of 0.24 cm for girls (AUC 0.834, 95%CI 0.772-0.895; 79% sensitivity and 90% specificity). The same cutoff value of 0.27 cm (AUC 0.858, 95% CI 0.820-0.896; 87% sensitivity, 79% specificity) was also suggested to discriminate healing for trials (Mayo 0-1) vs moderate to severe inflammation (Mayo 2-3) – supporting the challenge of defining accurate cutoff value for mild inflammation.

#### Crohn’s disease

Based on measured BWT of the colon, use of the cutoff of 0.30 cm detected 81/119 (68%) of inflamed segments, missing 38/119 (32%) and in the terminal ileum detected 22/30 (73%) and missed 8/30 (27%). In investigating the BWT cutoff value for CD, paired IUS–colonoscopy was available for 48 children with CD (258 paired segments from the colon and 45 from the terminal ileum) and 3 children without IBD (14 paired segments from the colon and 3 from the terminal ileum)—generating 272 paired IUS–colonoscopy segments from the colon and 48 from the terminal ileum. With good accuracy (AUC, 0.804; 95%CI 0.751-0.856), the BWT cutoff of 0.27 cm provided 68% sensitivity and 88% specificity for detecting colon inflammation. A cutoff of 0.24 cm for girls (AUC 0.835, 95%CI 0.695-0.867) showed 71% sensitivity and 92% specificity. Inflammation of the terminal ileum inflammation at colonoscopy was present in 36/48 segments. However, the low number of paired IUS–colonoscopy assessments from the terminal ileum resulted in poor accuracy of the ROC curve (AUC 0.571), preventing the determination of a reliable BWT cutoff for detecting inflammation.

Using segmental SES-CD scoring for colon without considering histological results to confirm inflammation (i.e., SES-CD 0-2 without ulcers vs the rest of scoring) did not change the suggested general cutoff value of 0.27 cm (AUC 0.840, 95% CI 0.788-0.891; 76% sensitivity and 85% specificity) either. Lower cutoff value of 0.24 cm (AUC 0.817, 95%CI 0.732-0.902; 79% sensitivity and 89% specificity) was also proposed for girls.

#### Very-early onset inflammatory bowel diseases

Based on measured BWT (including both colon and ileum), a cutoff value of 0.30 cm detected only 14/35 (40%) inflamed segments, missing the remaining 21/35 (60%). In 11 children (10 with VEO-IBD, 1 without IBD) who had IUS examination before the age of 6 years, paired BWT-bowel segment findings were available for 58 segments (51 from colon and 7 from terminal ileum). BWT poorly detected inflammation in the bowel segments of children under the age of 6 years old (AUC 0.667, 95%CI 0.526-0.808). For detecting inflammation in either the colon and/or terminal ileum, a tentative BWT cutoff value of 0.22 cm presented low sensitivity of 55% and specificity of 87%.

### Cutoff values for different degree of inflammation

**Figure 2.**
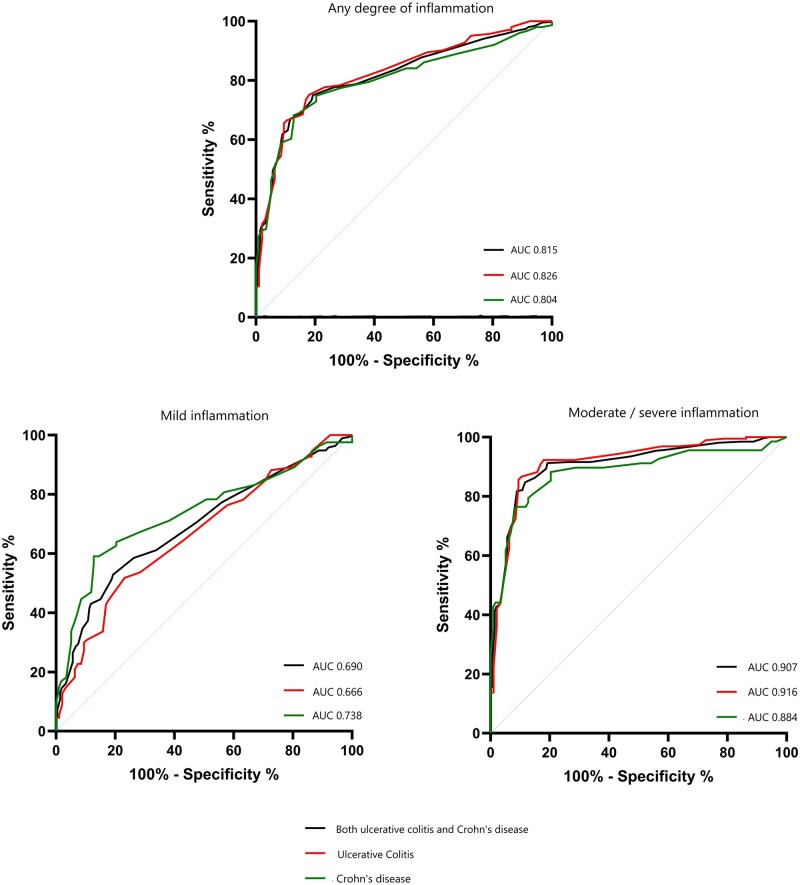
Receiver operating characteristic (ROC) curve with true positive rate (sensitivity) in relation to false positive rate (100% - specificity), presenting the accuracy of bowel wall thickness (BWT) for detecting inflammation in children ≥6 years old with ulcerative colitis (red), Crohn’s disease (green), or any type of IBD (black). Area under the curve (AUC) of 0.5 is displayed with blue line.

Based on measured BWT values of the colon, cutoff of 0.30 cm missed 143/446 (32%) of any degree of inflammation in both UC and CD, 92/162 (57) of mild inflammation, and 51/284 (18%) of severe inflammation. In ROC analysis, the ability of BWT to detect mild inflammation at colon segments of children ≥6 years old was poor (AUC 0.690, 95%CI 0.638-0.742) especially in UC (AUC 0.666, 95% CI 0.593-0.739), but fair in CD (AUC 0.738, 95% 0.664-0.811) (Figure 2). In mild CD, cutoff of 0.27 cm presented 59% sensitivity and 88% specificity. However, this threshold detected only 42% of mild colonic inflammation in UC and CD combined.

In moderate/severe inflammation of the colon segments, measured BWT showed that cutoff value of 0.30 cm detected 158/183 (86%) of inflammation in UC and 47/62 (76%) of inflammation in CD. BWT cutoff of 0.27 cm showed excellent accuracy (AUC 0.907, 95% CI 0.879-0.935) in detecting moderate-to-severe inflammation at colon segments of children ≥6 years old. This was true especially in UC (AUC 0.916, 95% CI 0.880-0.952), while in CD the accuracy was good (AUC 0.884, 95% CI 0.826-0.941). An optimal cutoff of 0.27 cm presented 87% sensitivity and 90% specificity for UC, and cutoff of 0.30 cm presented 77% sensitivity and 92% specificity for CD. When all paired BWT–bowel segment findings in children ≥6 years with UC, CD, or normal colon were analysed together, the cutoff of 0.27 cm showed excellent accuracy in detecting colon inflammation (AUC 0.908, 95% CI 0.880-0.936; 85% sensitivity, 88% specificity), with no need to differentiate UC from CD.

Based on measured BWT at colon, 0.30 cm cutoff value missed 112/377 (30%) of any degree of inflammation in children ≥6 years old. BWT cutoff of 0.27 cm showed good accuracy in detecting inflammation at colon segments (AUC 0.815, 95% CI 0.782-0.848; 67% sensitivity, 88% specificity), consistent across both diagnostic (AUC 0.802, 95% CI 0.743-0.861) and follow-up (AUC 0.823, 95% CI 0.782-0.856) IUS-colonoscopy pairs. The same threshold was also proposed for the terminal ileum in UC and CD (AUC 0.712, 95% CI 0.606-0.818; 61% sensitivity, 76% specificity).

### Cutoff values for different sex, weight, and age categories

During inflammation, girls have lower BWT than boys ­(Figure 1). Therefore, lower BWT cutoff value of 0.24 cm was proposed for girls (AUC 0.795, 95% CI 0.748-0.841; 73% sensitivity, 90% specificity), while boys could use the common BWT cutoff value of 0.30 cm (AUC 0.765, 95% CI 0.719-0.810; 61% sensitivity, 85% specificity) for detecting any degree of inflammation (Table 4).

**Table 4.** Optimal BTW cutoff value for detecting any degree of inflammation in the colon and/or terminal ileum of children with ulcerative colitis or Crohn's disease according to sex and weight.

Cutoff value	AUC	95% CI	Maximum Youden index		Proposed BWT cutoff value, cm		Sensitivity, %		Specificity, %	
**Based on sex**										
**Boys**	0.765	0.719-0.810	0.454		0.30		61		85	
**Girls**	0.795	0.748-0.841	0.624		0.24		73		90	
**Both**	0.784	0.752-0.816	0.494		0.27		63		86	
**Based on weight**										
**<40 kg**	0.748	0.690-0.806	0.453		0.24		71		74	
**≥40 kg**	0.801	0.758-0.843	0.513		0.28		63		89	
**Based on age**										
**<12 y**	0.737	0.681-0.794	0.461		0.24		71		76	
**≥12 y**	0.813	0.775-0.851	0.526		0.27		66		87	

Abbreviations: AUC, area under the curve; BWT, bowel wall thickness (with cutoff value for detecting inflammation at both colon and terminal ileum),

In children with PIBD, weight taken within 5 days before to 5 days after IUS were available for 714/792 (90%) BWT-bowel segment pairs. In children without PIBD, weight data taken within 11-36 days before IUS was available for 10/17 (59%) BWT-bowel segment pairs. Of these 724 weight data, 308 (38%) were <40 kg and 416 (51%) were ≥ 40 kg. Depending on clinical use, suggestive cutoff value for detecting any degree of inflammation in children <40 kg was 0.24 cm (AUC 0.748, 95% CI 0.690-0.806; 71% sensitivity, 74% specificity) and in those ≥ 40 kg was 0.28 cm (AUC 0.801, 95% CI 0.758-0.843, 63% sensitivity, 89% specificity). The same cutoff value of 0.24 cm (AUC 0.743, 95% CI 0.680-0.806) can also be used in children <35 kg, while cutoff of 0.27 cm (AUC 0.700. 95% CI 0.758-0.839) is suggested for children ≥ 35 kg. However, in ≥40 kg girls, the BWT cutoff of 0.24 cm (AUC 0.826, 95% CI 0.725-0.928; 81% sensitivity, 91 specificity 91%) was still applicable. BWT cutoff value based on age (<12 years vs ≥12 years) did not differ from cutoff value based on weight (<40 kg vs ≥40 kg).

## Discussion

IUS demonstrated excellent accuracy in detecting moderate to severe bowel inflammation in children aged 6 years and older. However, its accuracy was only fair in mild inflammation and poor in younger children with VEO-IBD. The BWT cutoff of 0.30 cm used in adults would miss over 30% of bowel inflammation in children. We propose an optimal BWT of 0.27-0.30 cm for general screening and monitoring of bowel inflammation in children ≥40 kg. This threshold is applicable for both UC and CD, across all colonic segments, and can be used in both diagnostic and follow-up IUS in children aged ≥6 years. However, a lower cutoff value of 0.23-0.25 cm for children <40 kg and/or girls should be strongly considered. In mild inflammation and in children under 6 years old, BWT was not consistently prominent, and IUS had poor accuracy although a tentative cutoff of 0.22 cm was observed. Clinicians should therefore interpret a “normal” appearing IUS with caution in young children or in those with mild symptoms, as inflammation may still be present despite subtle imaging findings.

Our study showed that BWT increased with degree on inflammation. However, establishing reliable BWT cutoff value for detecting mild inflammation in children ≥ 6 years old was challenging. Our study corresponded with a prospective study by Bremner et al (44 children and 153 bowel segments) and a cross-sectional study by Chavannes et al (33 children, 174 bowel segments).^13^^,^^14^ While Bremner and Chavannes used group comparison (mean BWT normal vs mildly inflamed bowel), we searched for an optimal BWT to predict inflammation using ROC curve with AUC. Both methods led to the same conclusion, confirming the reliability of our conclusion. Chavannes also attempted to find a predictive cutoff value for detecting any degree of inflammation (with AUC of 0.706), but did not discriminate mild and moderate/severe inflammation nor UC and CD.^14^ In contrast, The fair accuracy in detecting mild inflammation of colon may be explained by less colorectal submucosal fibrosis and muscularis mucosa thickening, which correlates with the degree of mucosal inflammation.^15^ Therefore, mild inflammation that has not proceeded to submucosal involvement may appear normal in the IUS.

IUS showed poor accuracy in detecting any degree of inflammation in children <6 years old. To date, no other studies have formally evaluated the accuracy of IUS in young children. This appears to be a disease-specific issue rather than a limitation of IUS as a cross-sectional imaging modality. For instance, a study comparing magnetic resonance enterography (MRE) with colonoscopy in children with very early-onset IBD (VEO-IBD) found that MRE detected only 29.4% of cases.^16^ Children with VEO-IBD are also known to exhibit different aetiopathogenesis than older children with pediatric-onset IBD. These include a greater chance of monogenic immunodeficiency, allergy, greater concern regarding immune dysregulation, T- or B-cell defects, phagocytic abnormalities, autoinflammatory conditions, and epithelial barrier dysfunction.^17^ Therefore, in mild inflammation or in children under 6 years, the additional information obtained from IUS should be interpreted with caution.

Kellar et al (98 children, 484 bowel segments) found no significant differences between BWT at different segments of colon during remission,^18^ as we did not find any segmental BWT differences during both absence and presence of inflammation. Therefore, similar cutoff values should be applicable in any segments of the colon. Kellar et al also noted that BWT may be affected by weight.^18^ In our study, BWT cutoff values was different in boys and girls, and in children <40 kg or ≥40 kg. This may reflect physiological differences emerging during or after puberty, as boys typically develop more lean body mass. These post-pubertal physiological changes may also be reflected as thicker layers of the bowel wall. Further studies to assess the role of pubertal status in BWT measurements are warranted.

Our data included a relatively small number of IUS–colonoscopy pairs from rectum, largely due to technical challenges in visualising the distal sigmoid and rectum posterior to the bladder. Therefore, in the diagnostic evaluation of IBD, IUS may miss UC manifested as proctitis only.^19^ However, an isolated proctitis is far less common a phenotype in children (5-15%) than adults with IBD and commonly clinically obvious with significant diarrhoea, per rectum blood loss. Such children usually have an elevated faecal calprotectin–features that would raise clinical suspicion for active IBD. Hence the limitations of IUS rectal assessment are not a major hurdle in pediatric IBD. In contrast, in children with reverse gradient colitis and more severe proximal inflammation, full colonoscopy may be harder to perform and may be unsuccessful in 5% of children. In such scenarios, IUS offers a valuable, noninvasive means of assessing inflammation in segments of the colon that may be inaccessible or risky to evaluate endoscopically.^19^ Therefore, IUS and colonoscopy should be viewed as complementary tools, each offering unique strengths that, when used together, enhance the overall assessment and management of pediatric IBD.

### Strength and limitations

A key strength of this study is its large pediatric cohort, comprising 136 children and 809 paired BWT measurements across different bowel segments, collected over more than five years of IUS investigations. All IUS examinations were performed by a single experienced operator, thereby eliminating interobserver variability—a common confounding factor in ultrasound studies. Notably, this is one of the few studies to investigate BWT cutoff values exclusively in children. Furthermore, 73% of IUS studies were conducted prior to the corresponding colonoscopy, effectively blinding the IUS and minimising bias. For the remaining cases, the interval between colonoscopy and IUS was no more than 7 days, reducing the likelihood of significant mucosal improvement that could confound interpretation. In children aged ≥6 years, we also performed separate analyses for ulcerative colitis (UC) and Crohn’s disease (CD) to explore potential differences in BWT cutoff values. Interestingly, the resulting thresholds were comparable for both conditions, supporting the applicability of a unified cutoff in this age group.

In this retrospective study, we relied on existing clinical data, providing imitations. Approximately 30% of BWT values stated as “normal” without specific measurements at the clinical report had to be imputed (Table S1). However, our rigorously applied and validated imputation approach, using values from the most clinically similar segment, is a widely accepted method in clinical research and has been validated in similar contexts.^20^

In conclusion, IUS demonstrated excellent accuracy in detecting moderate to severe intestinal inflammation in children aged 6 years and older. A BWT cutoff value of 0.27-0.30 cm should prompt clinical suspicion for active inflammation. However, even lower thresholds of 0.23-0.25 cm should be strongly considered in girls and children <40 kg. IUS in children under the age of 6 years commonly misses or underestimates IBD inflammation and findings often considered as “normal” is insufficient to rule out IBD in this age group.

## Supplementary Material

izaf298_Supplementary_Data

## Data Availability

The data underlying this article will be shared on reasonable request to the corresponding author.
