# Supplementary material for: Bowel Wall Thickness Cutoff Value for Assessing Inflammatory Bowel Disease Activity Using Intestinal Ultrasonography in Children
Source: Inflamm Bowel Dis. 2025 Dec 8;32(4):730–9. doi: 10.1093/ibd/izaf298 (PMC13046047; doi:10.1093/ibd/izaf298)
Supplement: izaf298_Supplementary_Data [file izaf298_supplementary_data.docx]

| **Age at IUS examination, years** | **Mean BWT (SD)** | | | |
| --- | --- | --- | --- | --- |
|  | **Boys** | **Girls** | **TOTAL** |  |
| <6 (n=3+10) | 0.20 (0.05) | 0.18 (0.07) | 0.18 (0.06) |  |
| 6 (n=1+1) | 0.12 | 0.21 | 0.17 (0.06) |  |
| 7 (n=0) | n/a | n/a | n/a |  |
| 8 (n=3+0) | 0.15 (0.05) | n/a | 0.15 (0.05) |  |
| 9 (n=2+5) | 0.17 (0.00) | 0.24 (0.03) | 0.22 (0.04) |  |
| 10 (n=2+4) | 0.22 (0.00) | 0.24 (0.08) | 0.23 (0.06) |  |
| 11 (n=17+17) | 0.21 (0.06) | 0.22 (0.08) | 0.22 (0.07) |  |
| 12 (n=7+8) | 0.17 (0.02) | 0.20 (0.05) | 0.19 (0.04) |  |
| 13 (n=n=9+7) | 0.31 (0.19) | 0.17 (0.01) | 0.25 (0.16) |  |
| 14 (n=32+21) | 0.23 (0.06) | 0.18 (0.06) | 0.21 (0.07) |  |
| 15 (n=4+14) | 0.16 (0.09) | 0.17 (0.02) | 0.17 (0.04) |  |
| 16 (n=3+5) | 0.20 (0.00) | 0.19 (0.04) | 0.19 (0.03) |  |
| 17 (n=0+5) | n/a | 0.22 (0.00) | 0.22 (0.00) |  |

**Supplementary Table 1.** Mean bowel wall thickness (BWT) of 180 imputated missing “normal” values obtained from adjacent or other segments of colon from the same patient at the same IUS session, also reported as “normal” in the clinical note by the operator.
